# Sohlh2 suppresses epithelial to mesenchymal transition in breast cancer via downregulation of IL-8

**DOI:** 10.18632/oncotarget.10355

**Published:** 2016-06-30

**Authors:** Shufang Ji, Wenfang Zhang, Xiaoli Zhang, Chunyan Hao, Aijun Hao, Qing Gao, Hongying Zhang, Jinhao Sun, Jing Hao

**Affiliations:** ^1^ Key Laboratory of The Ministry of Education for Experimental Teratology, Department of Histology and Embryology, School of Medicine, Shandong University, Jinan 250012, PR China; ^2^ Department of Pathology, School of Medicine, Shandong University, Jinan 250012, PR China; ^3^ Department of Biology, Jinan Vocational College of Nursing, Jinan 250000, PR China; ^4^ Department of Human Anatomy, School of Medicine, Shandong University, Jinan 250012, PR China

**Keywords:** Sohlh2, IL-8, EMT, metastasis, breast cancer

## Abstract

Breast cancer is one of the deadliest cancers worldwide due to its strong metastasis to other organs. Metastasis of breast cancer involves a complex set of events, including epithelial-mesenchymal transition (EMT) that increases invasiveness of the tumor cells. We previously identified sohlh2 is a tumor suppressor in the pathogenesis of ovarian cancer. However, the functions of sohlh2 in breast cancer cell migration and invasion remain unknown. Here we report a novel sohlh2/IL-8 signaling pathway in the invasive breast cancer. We observed sohlh2 expression was downregulated in the metastatic breast cancer. Ectopic sohlh2 expression in breast cancer cells reduced EMT and inhibited cell migration and invasion *in vitro*, and metastasis *in vivo*. Moreover, the depletion of sohlh2 induced the opposite effects to ectopic sohlh2 expression. RNA-Seq data from a sohlh2 knockdown breast cancer cell line showed that after sohlh2 depletion, the mRNA level of interleukin 8 (IL-8) was significantly increased in these cancer cells, which consequently increased secretion of IL-8 protein. Using chromatin immunoprecipitation and reporter assays, we demonstrated that sohlh2 bound to IL-8 promoter and repressed its activities. The enhanced migration and invasion in sohlh2 -ablated MCF-7 cells were blocked by knockdown of IL-8 expression, while exogenous IL-8 neutralized the anti-migratory and invasive activities of sohlh2 in MDA-MB-231cells. Overall, these results demonstrate that sohlh2 functions as a tumor metastasis suppressor via suppressing IL-8 expression in breast cancer.

## INTRODUCTION

Breast cancer is one of the leading causes for cancer death in females worldwide [1]. The majority of breast cancer-related deaths are caused by metastatic progression, which is a complex process involving a succession of cell biological events [2, 3]. Currently, treatment options for metastatic breast cancers are limited and ineffective. Therefore, tremendous efforts have been made to understand the mechanisms how metastasis occurs, which will help to develop more rational approaches for treatments of metastatic breast cancers. However, how metastases are formed remains less understood. Mounting evidence shows that in epithelial cancers, including breast cancers, induction of epithelial–mesenchymal transition (EMT) is a major event that provides the mobility to cancer cells to generate metastases [4].

EMT is characterized by the loss of epithelial characteristics and acquisition of a mesenchymal phenotype, which confers the ability for cancer cells to invade adjacent tissue and disseminate to distant sites [5]. The process of EMT is featured with the loss of epithelial markers, such as E-cadherin and occludin, and acquisition of mesenchymal markers, such as N-cadherin and vimentin. Many factors can stimulate the EMT process. A group of transcription factors have been demonstrated to be capable of orchestrating EMT in cancer progression, including Snail, Slug, and ZEB2/SIP1 [6, 7]. Hence clarification of EMT mechanisms can greatly benefit our understanding to tumor metastases.

A spermatogenesis and oogenesis specific basic helix-loop-helix (bHLH) transcription factor, sohlh2, is one of important transcription factors in large bHLH family [8, 9]. bHLH proteins play critical roles in many physiological processes including cellular differentiation, cell cycle arrest and apoptosis [10]. The conserved bHLH domain is involved in homo- or hetero-dimerization to form a functional transcriptional unit that binds to the canonical E-Box response element (CANNTG) in the promoter of many genes [10]. In human, our previous data showed that high level of sohlh2 expression was observed in various normal tissues, especially epithelial tissue. We also demonstrated that overexpression of sohlh2 resulted in the inhibition of ovarian cancer cell proliferation, migration and invasion [11, 12]. Moreover, targeted expression of sohlh2 inhibited ovarian cancer growth and metastasis *in vivo* [11, 12]. These findings suggest that sohlh2 acts as a novel tumor suppressor.

Interleukin 8 (IL-8), alternatively known as CXCL8, is a pleiotropic chemokine involved in variety of pathophysiological processes. It has been shown to play an important role in human cancers by modulating metastasis and angiogenesis [13]. IL-8 activates two cell-surface, G protein-coupled receptors (CXCR1 and CXCR2) in multiple intracellular signaling pathways [14]. Increased expression of IL-8 and/or its receptors has been observed in a number of cancers including breast cancer [15, 16]. Increased level of serum IL-8 has been reported in metastatic breast cancers, which correlates with early dissemination and survival [17]. Moreover, it has been demonstrated that IL-8 is involved in the regulation of EMT process [18, 19].

In this study, we demonstrated that sohlh2 overexpression inhibited EMT and metastasis in breast cancer cells. Conversely, sohlh2 silencing induced EMT in transformed and malignant human mammary epithelial cells, resulting in enhancement of migration and invasion *in vitro*. These functional effects of sohlh2 were achieved by directly binding to IL-8 promoter and controlling IL-8 transcriptional expression. Our findings provide novel mechanistic insights of sohlh2 in breast cancer metastasis and a reasonable explanation for our clinical observation that low sohlh2 expression is correlated with breast cancer metastasis, suggesting that sohlh2 may serve as a potential therapeutic target for advanced breast cancers.

## RESULTS

### Sohlh2 reduction in breast cancer correlates with metastasis

**Figure 1:**
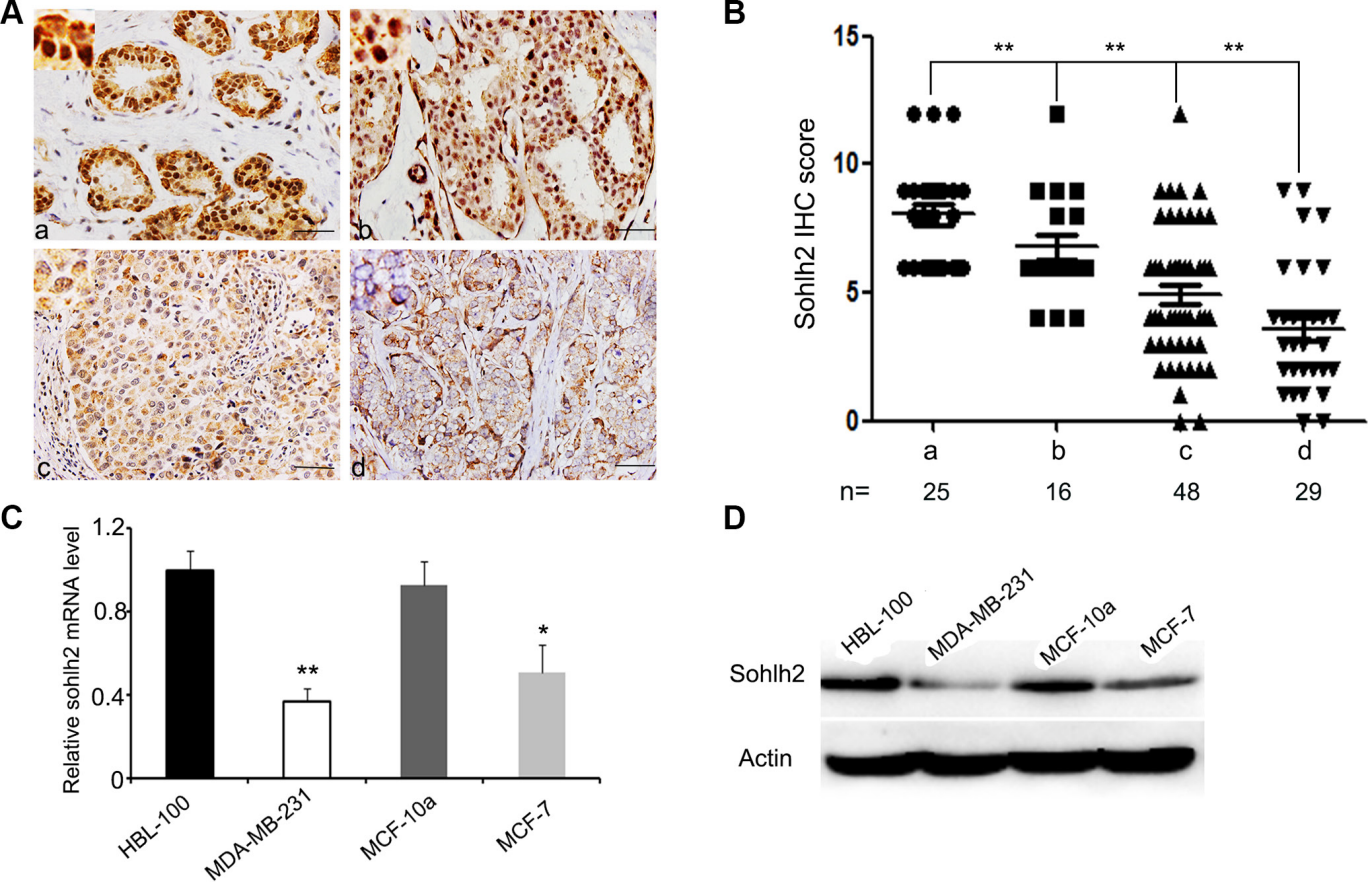
Reduced expression of sohlh2 is correlated with the metastasis of breast cancer (**A)** Immunohistochemical staining of Sohlh2 in adjacent tissues of breast cancer (a), intraductal carcinoma *in situ* (b), breast cancer without or with metastasis (c, d). A representative image from each group is shown. Scale bar = 50 μm. (**B)** Intensity of sohlh2 staining was scored from 0 to 12. Graph representing the average intensity of sohlh2 staining for adjacent tissues of breast cancer (a) versus intraductal carcinoma *in situ* (b), breast cancer without or with metastasis (c, d). (**C, D)** The mRNA and protein expression of sohlh2 were analyzed by qPCR (C) and Western blot (D) in the transformed mammary epithelial cell lines (HBL-100, MCF-10a) and breast cancer cell lines (MDA-MB-231, MCF-7). β-actin was used as a loading control. *P < 0.05; **P < 0.01.

Sohlh2 expression was examined in 77 cases of primary human invasive ductal breast cancer specimens, 16 cases intraductal carcinoma *in situ*, and 25 cases adjacent tissues of breast cancer by immunohistochemitry (IHC) assay. Sohlh2 expression was observed in all (100%) adjacent tissues, all (100%) intraductal carcinoma *in situ*, and in 47 of 77 (61.04%) of invasive ductal carcinoma. Sohlh2 immunostaining was both nuclear and cytoplasmic (Figure 1A). Sohlh2 expression was significantly lower in invasive ductal carcinoma with (*n* = 29) and without metastasis (*n* = 48) compared to intraductal carcinoma *in situ* (*n* = 16) and adjacent tissues (*n* = 25), with a median IHC-score 8.04 for adjacent tissues, 6.76 for intraductal carcinoma *in situ*, 4.96 for breast cancer without matastasis, and 3.6 for breast cancer with matastasis, respectively (*p < 0.01*; Figure 1B). Moreover, sohlh2 expression was negatively associated with lymph node metastasis and distant metastasis in breast cancer (*p <* 0.01, Figure 1B).

We sought to examine the mRNA and protein levels of sohlh2 expression in human breast cancer cell lines (MDA-MB-231, MCF-7) and transformed mammary epithelial cell lines (HBL-100, MCF-10a) by qPCR and Western blot analysis. As shown in Figure 1C and 1D, sohlh2 mRNA and protein were lowly expressed in human breast cancer cell lines, especially in MDA-MB-231 cells, compared with those in HBL-100 and MCF-10a cells. These data strongly suggest that sohlh2 expression is significantly suppressed in human breast cancer cells.

### Sohlh2 represses migratory and invasive capacities of breast cancer cells *in vitro*

**Figure 2:**
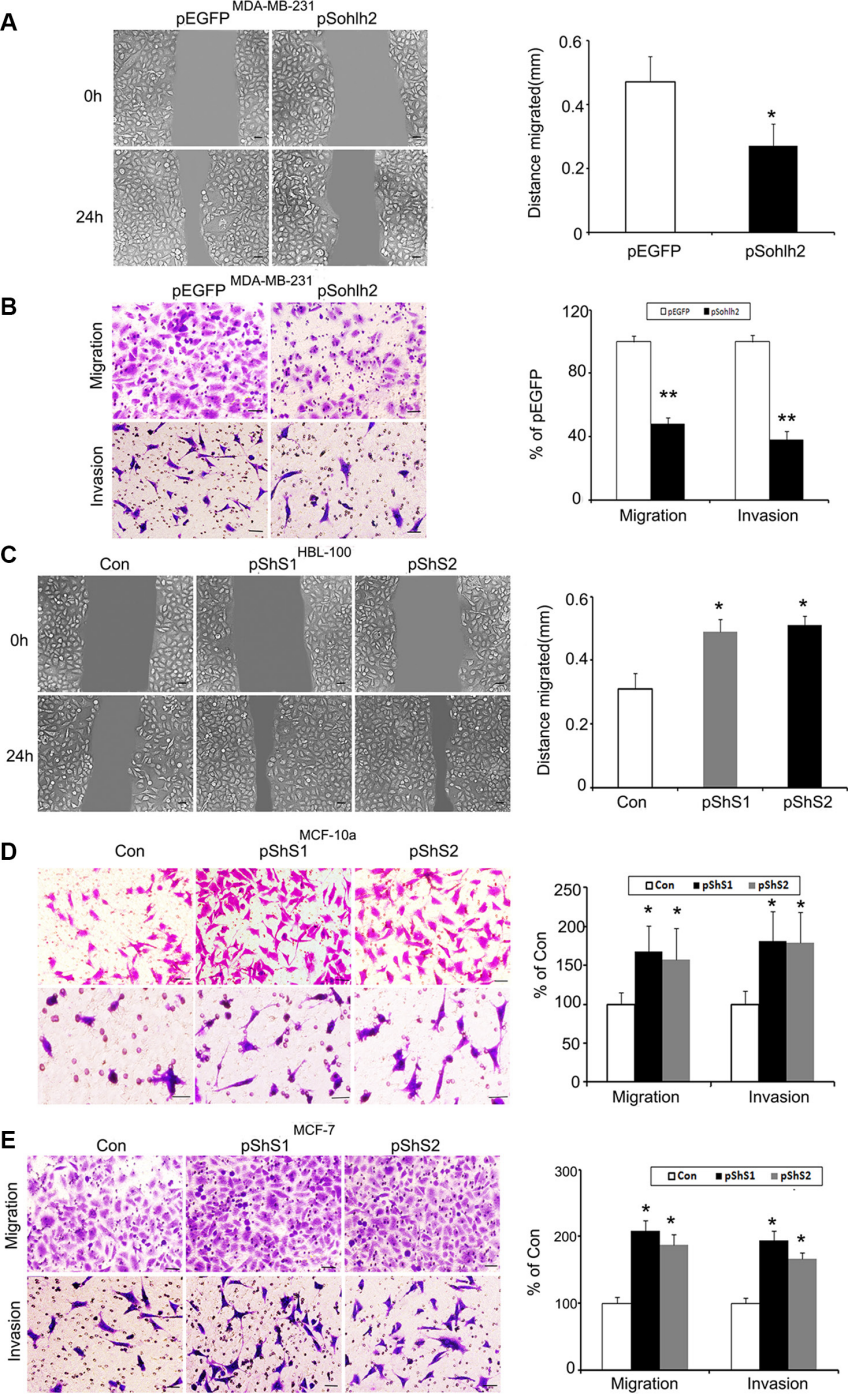
Sohlh2 expression reduces breast cancer cell migration and invasion (**A**) Sohlh2 overexpression decreases cell migration in MDA-MB-231 cells. (**B**) Sohlh2 overexpression inhibits cell migration and invasion in MDA-MA-231 cells. (**C**) Sohlh2 knockdown increases cell migration in HBL100 cells. (**D**) Sohlh2 knockdown increases cell migration and invasion in MCF-10a cells. (**E**) Sohlh2 knockdown increases cell migration and invasion in MCF-7 cells. The representative images were shown on the left and the quantitative analysis was shown on the right. The cell migration distance (in A and C) was quantified by wound healing assay, while quantification of migrated cells through the membrane and invaded cells through Matrigel of each cell line (in B, D, E) was carried out by transwell assays. Scale bars indicate 100 μm (A and C) or 50 μm (B and D). All results are obtained from three independent experiments. *P < 0.05; **P < 0.01.

The effect of sohlh2 on cell migration was assessed by wound healing assay. Sohlh2 overexpression in MDA-MB-231 cells significantly slowed down closure of the wound area compared with control cells (Figure 2A). This result was further verified by Transwell assay (Figure 2B). Sohlh2 overexpression in MDA-MB-231 cells showed a less degree of invasion through Matrigel (Figure 2B). Conversely, sohlh2 silencing dramatically enhanced the migratory and invasive capacities of HBL100, MCF-10a and MCF7 cells (Figure 2C–2E). These results indicate that sohlh2 represses migratory and invasive behaviors in breast cancer cells.

### Sohlh2 inhibits metastasis *in vivo*

**Figure 3:**
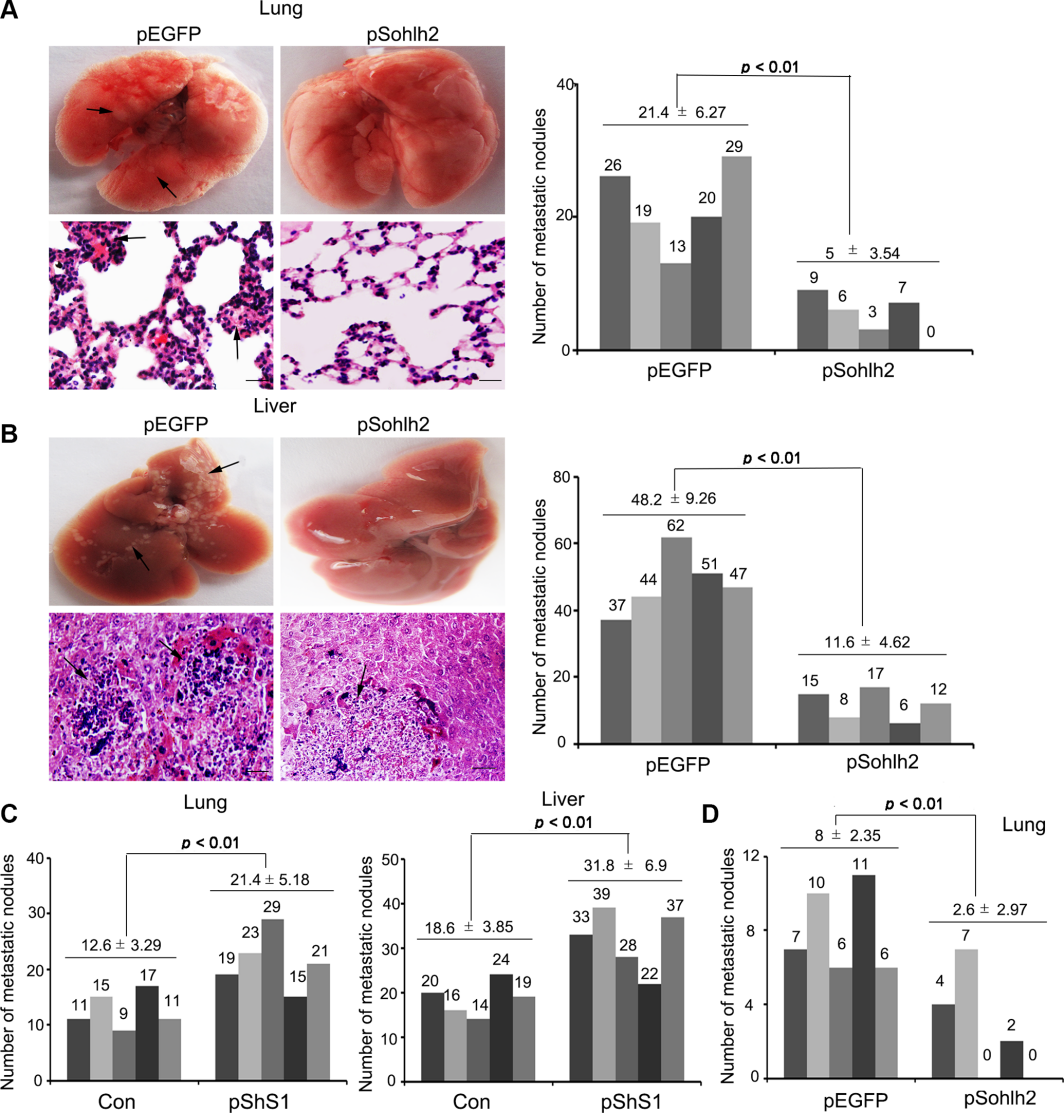
Sohlh2 inhibits breast cancer metastasis in mice Sohlh2–expressing (sohlh2-silencing) or parental cells were injected into BALB/c nude mice via tail vein. The mice were sacrificed after 8 weeks, and tumor colonies in the lung and liver were examined. (**A**) Representative images of lung samples and sections with H&E staining (right), metastasis nodules formed by parental and sohlh2–expressing MDA-MB-231 cells were quantified (left). (**B**) Representative images of liver samples and sections with H&E staining at 8 wk after injection were shown (right), metastasis nodules formed by parental and sohlh2–expressing MDA-MB-231 tumors were quantified (left). (**C**) Metastasis nodules in lungs and livers formed by parental and sohlh2–silencing MCF-7 cells were quantified. (**D**) Sohlh2–expressing or parental cells were injected in mammary pad of BALB/c nude mice. Spontaneous metastasis nodules in lungs formed by parental and sohlh2–expressing MDA-MB-231cells were quantified. Arrows show metastasis nodules. Scale bars indicate 50 μm.

To extend our *in vitro* observations, we investigated whether sohlh2 can decrease metastatic capacity of breast cancer cells *in vivo*. The functional relevance of sohlh2 for metastasis *in vivo* was assayed. Sohlh2 overexpression in MDA-MB-231 cells dramatically decreased, while sohlh2 knockdown in MCF-7 cells significantly increased the number of metastatic tumors in the lungs and livers via tail vein injection (Figure 3A–3C). The spontaneous tumor dissemination was also evaluated in the lungs by injection MDA-MB-231 cells in the mammary fat pad. As shown in Figure 3D, sohlh2 overexpression dramatically decreased the number of metastatic tumors in the lungs. These results indicate that sohlh2negatively regulates the metastasis of breast cancer cells. Therefore, the *in vivo* results further validate the critical role of sohlh2 in breast cancer metastasis.

### Sohlh2 reduces EMT

**Figure 4:**
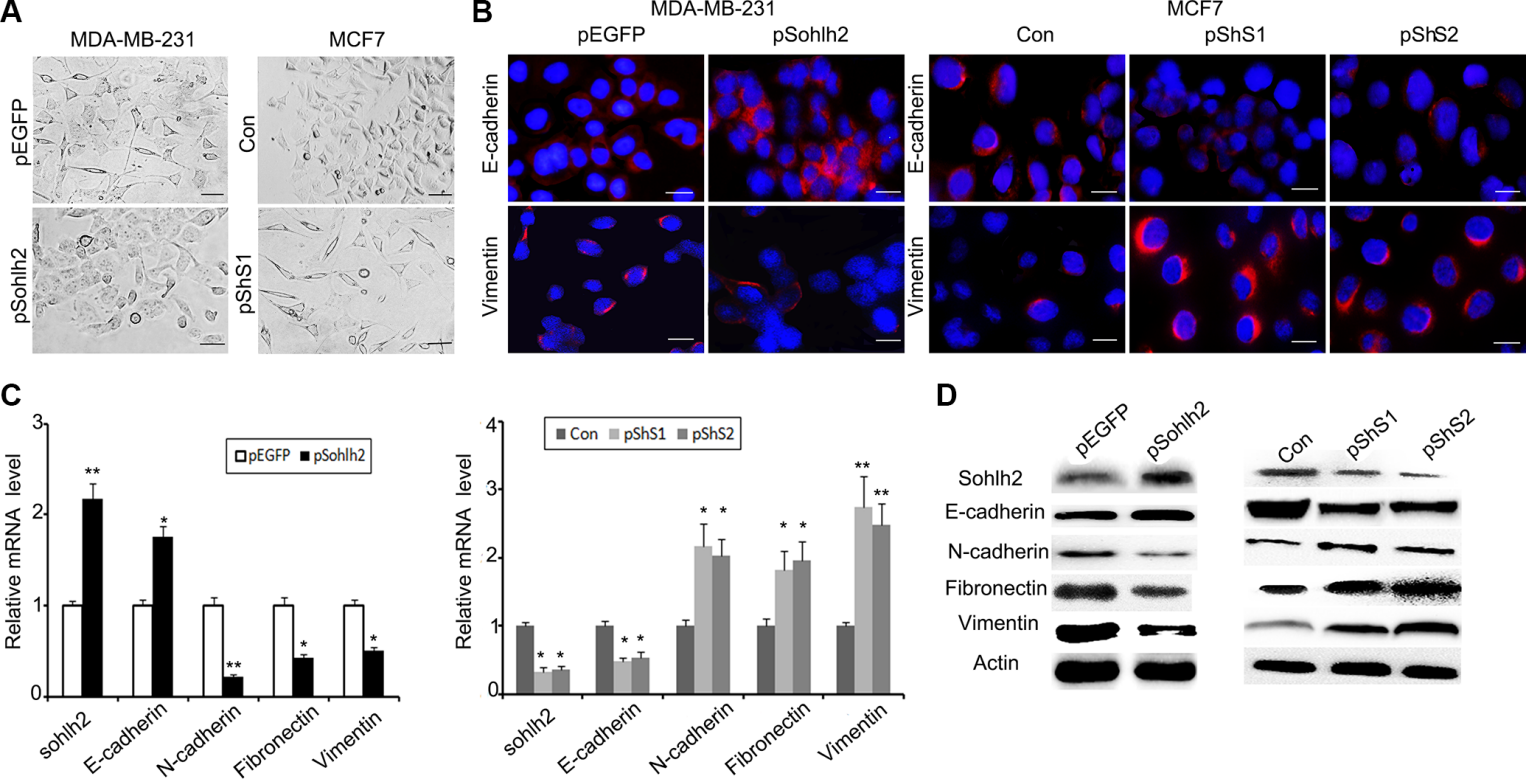
Sohlh2 suppresses EMT in breast cancer cells (**A**) Phase contrast photographs show that pShS1-transfected MCF7 cells display fibroblastic morphology with increased cell spreading, while pSohlh2-transfected MDA-MB-231 cells display epithelial morphology. Scale bar = 100 μm. (**B**) Immunostaining of E-cadherin and vimentin in MDA-MB-231 and MCF-7 cells. The nuclei were stained by DAPI. Scale bar = 50 μm. (**C**) The mRNA expression of EMT markers including E-cadherin, N-cadherin, Fibronectin and vimentin in sohlh2-overexpressing MDA-MB-231 cells or sohlh2-ablated MCF-7 cells was examined by qPCR. (**D**) Cell lysates were prepared and the protein expression of EMT markers was analyzed by immunoblotting. β-actin was used as an internal control. All results are obtained from three independent experiments. *P < 0.05; **P < 0.01.

EMT plays a critical role in promoting metastasis of breast cancer. To investigate whether sohlh2 is involved in EMT in breast cancer cells, the expression of EMT biomarker proteins was examined. We showed that sohlh2 overexpression in MDA-MB-231 cells significantly increased the expression of epithelial marker E-cadherin but decreased the levels of mesenchymal markers (N-cadherin, fibronectin, and vimentin) (Figure 4A– 4D). Moreover, the expression of EMT biomarker mRNAs correlated with their corresponding protein levels (Figure 4C), suggesting that sohlh2 regulates the expression of EMT biomarkers at the transcript level. Conversely, Sohlh2 silencing in MCF7 cells exhibited fibroblastic properties (Figure 4A). Sohlh2 silencing significantly decreased the level of E-cadherin but increased the level of N-cadherin, fibronectin, and vimentin (Figure 4B–4D). Thus, these findings support that sohlh2 plays an important role in the regulation of EMT/MET plasticity in breast cancer cells.

### Sohlh2 downregulates IL-8 expression

**Figure 5:**
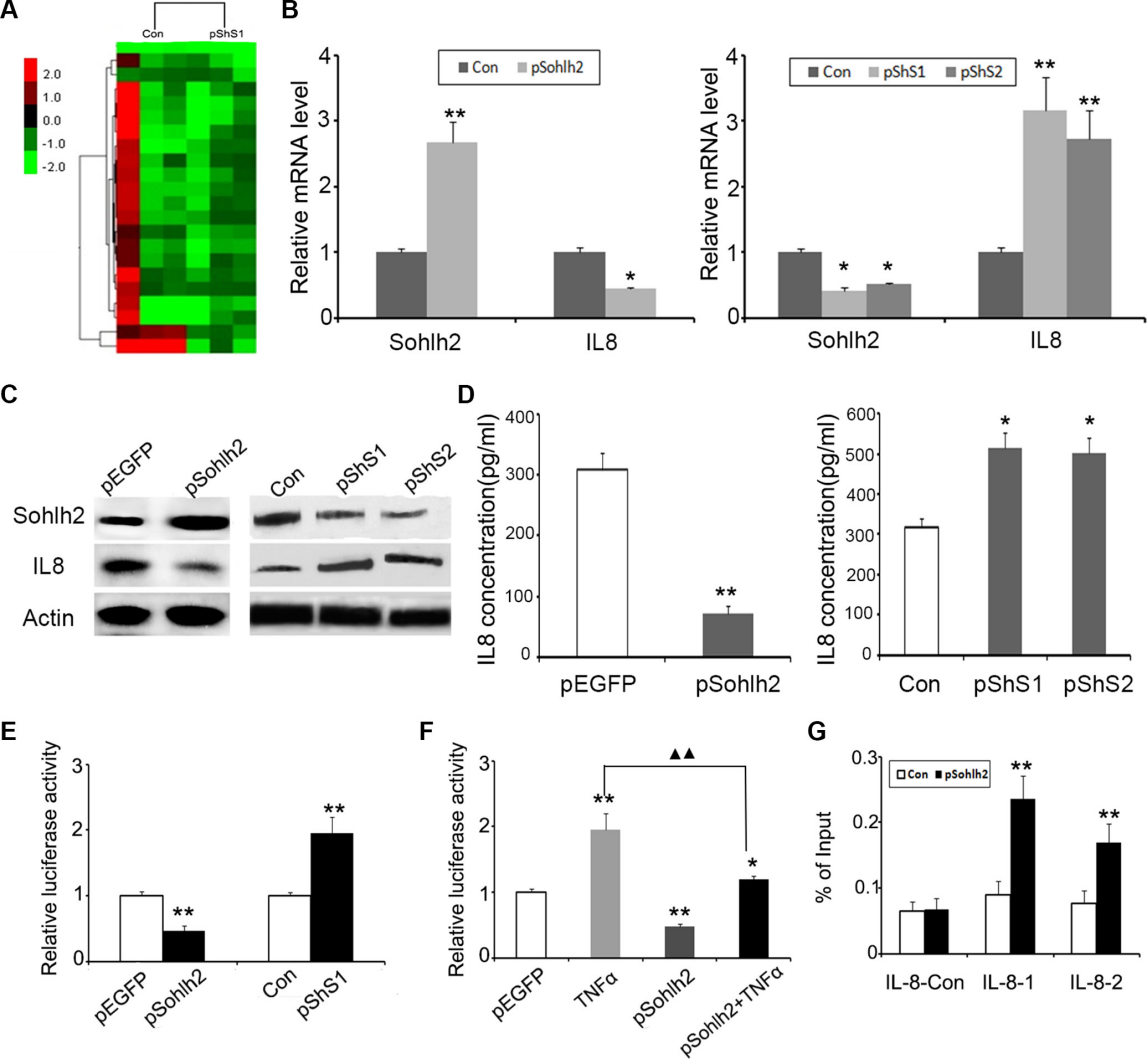
Sohlh2 regulates IL-8 expression (**A**) Supervised hierarchical clustering of differentially expressed genes after sohlh2 knockdown in MCF-7 cells. (**B, C**) The mRNA (B) and protein (C) expression of IL-8 and sohlh2 in breast cancer cell lines. β-actin was used as a loading control. (**D**) Measurement of IL-8 secretion in sohlh2-overexpressing MDA-MB-231 and sohlh2-ablated MCF-7 cells by ELISA. E. Inhibition of pGL4-IL-8 luciferase activity by sohlh2 overexpression in MDA-MB-231 cells, and activation of pGL4-IL-8 luciferase activity by sohlh2 silencing in MCF-7 cells. (**F**) Sohlh2 partially repressed the activation of IL-8 promoter caused by TNFα. ▲▲*P* < 0.01. (**G**) ChIP assay was carried out in sohlh2-overexpressing MDA-MB-231 cells. qPCR was performed to determine sohlh2 occupied abundance on IL-8 promoter with 2 primer pairs spanning E-Motifs and one control primer. qPCR results showed that sohlh2 bound to the specific regions of IL-8 promoter. Results represent mean ± SD from three independent experiments. *P < 0.05; **P < 0.01.

To better understand the mechanisms how sohlh2 is involved in breast cancer progression, we performed gene expression profiling sohlh2-ablated and control MCF7 cells. RNA-sequence analysis identified a list of differentially expressed genes after sohlh2 deletion, including upregulation of IL-8 (Figure 5A; Supplementary Table S1). It has been shown that IL-8 is involved in the regulation of EMT [18, 19]. These evidence prompts us to hypothesize that sohlh2 exerts these functions possibly via downregulation of IL-8. To test this, we first determined whether IL-8 is a downstream target of sohlh2 in breast cancer cells. Expression of IL-8 in the cells with altered expression was further evaluated by qPCR, Western blot and ELISA. Sohlh2 overexpression in MDA-MB-231 cells significantly decreased IL-8 mRNA and protein expression, whereas sohlh2 silencing in MCF7 cells significantly increased IL-8 mRNA and protein expression (Figure 5B–5D). These data suggest IL-8 expression is regulated by sohlh2 at transcriptional level.

### Direct inactivation of IL-8 gene by sohlh2

Sohlh2 functions as a bHLH transcription factor to regulate gene expression directly. To elucidate how sohlh2 inhibits IL-8 expression, the sequences in IL-8 promoter region were analyzed. Ten putative sohlh2-binding sites, E-boxes with a motif sequence CANNTG, were identified in the proximal (−2000 to +100 base pairs) promoter region of IL-8. This suggests that sohlh2 may down-regulate IL-8 expression by binding directly to the IL-8 promoter. We next performed reporter assay to investigate whether the IL-8 promoter is inactivated by sohlh2. IL-8 promoter activity was significantly repressed in sohlh2-overexpressing MDA-MB-231 cells, while IL-8 promoter activity was significantly stimulated in sohlh2 knockdown MCF-7 cells (Figure 5E). It is known that IL-8 promoter is directly activated by TNFα / NFκB signaling pathway. As shown in Figure 5F, sohlh2 partially blocked the activation of IL-8 promoter caused by TNFα. Chromatin immunoprecipitation assay identified the presence of the interacting IL-8 promoter fragments with sohlh2 in MDA-MB-231 cells (Figure 5G).

### IL-8 compensates the negative effects of sohlh2 on EMT, migration, and invasion in breast cancer cells

**Figure 6:**
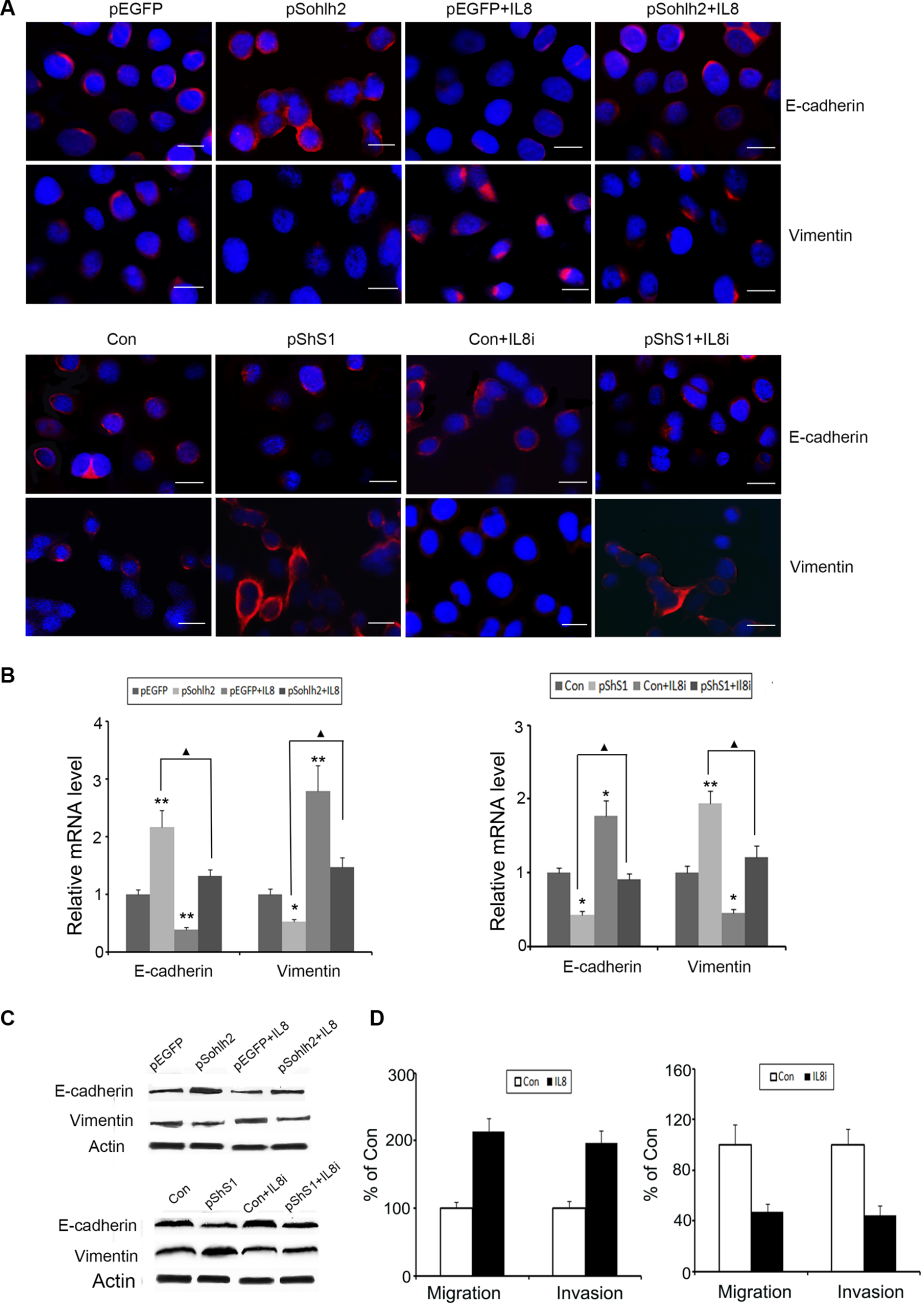
IL-8 is required for sohlh2-mediated EMT, migration and invasion (**A–C**) IL-8 decreased E-cadherin expression but enhanced vimentin expression in sohlh2-overexpressing MDA-MB-231 cells. IL-8 Silencing induced the opposite effects in sohlh2-ablated MCF-7 cells. (**D**) IL-8 enhanced transwell migration and Matrigel invasion in sohlh2-overexpressing MDA-MB-231 cells. IL-8 silencing inhibited transwell migration and Matrigel invasion in sohlh2-ablated MCF-7 cells. Scale bar = 50 μm. All results are obtained from three independent experiments. *P < 0.05; **P < 0.01.

To test whether sohlh2-reduced metastatic capacity was mediated by downregulation of IL-8, human recombinant IL-8 was used in sohlh2-overexpressing MDA-MB-231 cells, and short hairpin RNAs (shRNAs) of IL-8 were used in sohlh2-ablated MCF7 cells. As expected, IL-8 significantly suppressed E-cadherin expression but increased vimentin expression at mRNA and protein levels in sohlh2-overexpressing MDA-MB-231 cells (Figure 6A–6C). Conversely, knockdown of IL-8 in sohlh2-ablated MCF7cells resulted in elevated E-cadherin expression and decreased vimentin expression at mRNA and protein levels (Figure 6A–6C). Moreover, IL-8 treatment significantly enhanced the reduced migratory and invasive capacities in sohlh2-overexpressing MDA-MB-231 cells, while IL-8 silencing significantly suppressed the elevated migratory and invasive capacities in sohlh2-ablated MCF7 cells (Figure 6D).

### Converse correlation of sohlh2 and IL-8 expression in breast cancer tissues

**Figure 7:**
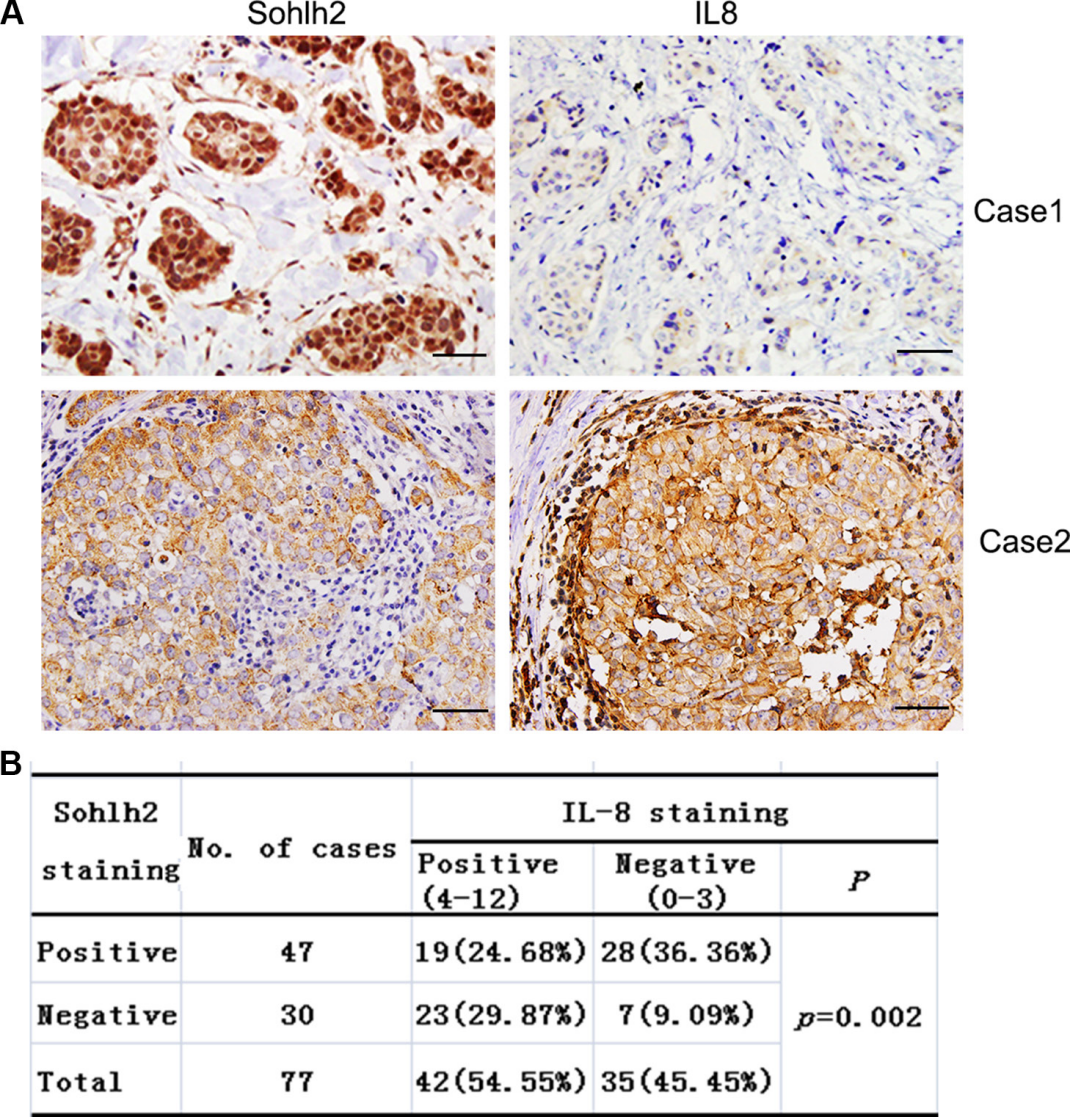
Correlation analysis of Sohlh2 expression with IL-8 in breast cancer specimens (**A)** Immunohistochemical staining of Sohlh2 and IL-8 in adjacent sections from the same patient. Scale bar = 50 μm. (**B)** Chi-square analysis of the correlation between Sohlh2 levels and IL-8 levels in breast cancer specimens.

It has been reported that breast cancer patients who have abundant IL-8 protein expression are more likely to develop distant metastases and have a poor survival outcome [14–16]. To investigate the correlation between sohlh2 and IL-8 expression in breast cancer tissues, immunohistochemistry analysis was then performed on 77 paraffin-embedded breast cancer tissue samples. Two cases with typical sohlh2 and IL-8 staining are shown in Figure 7A. A significant correlation between sohlh2 and IL-8 expression (*P* = 0.002, Figure 7B) was found. IL-8 expression was negatively correlated with sohlh2 expression in breast cancer tissues. These results suggest the presence of a sohlh2/IL-8 signaling in breast cancer samples.

## DISCUSSION

In the current study, we observed that the expression of sohlh2, a bHLH transcription factor, was down-regulated in breast cancer. The reduction in sohlh2 expression paralleled the invasion and metastasis of breast cancer. Invasive and migratory capabilities of breast cancer cell lines were significantly enhanced after sohlh2 knockdown, but significantly inhibited after sohlh2 overexpression. Sohlh2 overexpression also repressed the metastasis of breast cancer cells *in vivo*. A recent study by our group has shown that sohlh2 is repressed in ovarian carcinoma [11, 12]. Sohlh2 reduction shows strong associations with the metastasis and overall survival of ovarian cancer patients. Collectively, these results indicate sohlh2 is a novel tumor metastatic repressor.

EMT is associated with loss of epithelial features and gain of mesenchymal characteristics, resulting in increased invasive potential in epithelial cancer [5]. This study addressed the importance of sohlh2 in EMT and investigated the underlying molecular mechanisms. We found that sohlh2 siRNA induced elongated and spindle-like morphology (mesenchymal phenotype) and significant alteration of EMT markers expression in MCF-7 cells. Conversely, sohlh2 overexpression in MDA-MB-231 cells resulted in gain of epithelial characteristics and loss of mesenchymal characteristics, suggesting that sohlh2 is actively involved in the EMT process in breast cancer cells. Many bHLH transcription factors, such as Twist1, TCF4, DEC1, have been reported to act as strong promoters of EMT and metastatic spread in many human carcinomas [20–24]. However, a recent study revealed that a new bHLH transcription factor, E2A, is involved in suppressing the EMT process in colon cancer [25], which is consistent with our findings of repressed EMT by sohlh2. To the best of our knowledge, this is the first report suggesting the novel role of sohlh2 in the inhibition of EMT and metastasis in breast cancer cells.

Our study revealed a critical role of sohlh2/IL-8 signaling in EMT. In RNA-sequence results, IL-8 is the most upregulated gene in sohlh2-ablated MCF7 cells. The inflammatory mediators, such as TNFα, TGFβ and IL-6, and snail have been implicated in the initiation of EMT [26–28]. IL-8 expression was elevated in both invasive cancer cells [29, 30] and the sera of patients with aggressive cancers [31, 32]. Previous studies have reported that IL-8 is involved in the regulation of EMT process [18, 19, 33, 34]. Here we showed that sohlh2 inhibited IL-8 expression and EMT in breast cancer cells. Ectopic IL-8 addition in sohlh2-overexpressing MDA-MB −231 cells blocked the inhibitory effect of sohlh2 on EMT. Moreover, the enhanced EMT induced by sohlh2 knockdown was abolished by IL-8 silencing in breast cancer cells. This study indicates that IL-8 induction after sohlh2 knockdown is critical for EMT and thus provides a plausible molecular mechanism contributing to breast cancer aggressiveness.

Although IL-8 expression is regulated at both posttranscriptional and transcriptional levels, transcriptional regulation is of major importance for most stimuli, such as IL-1, TNFa, hypoxia, and acidic pH induces IL-8 expression in most types of cells [35–39]. In MDA-MB −231ER-negative breast cancer cells, IL-8 expression is positively regulated by NFκB, and synergistically cooperated by AP-1 and C/EBP transcription factors [40]. Indeed, we found that sohlh2 negatively regulated IL-8 expression at transcriptional level. Luciferase reporter and CHIP assays revealed that sohlh2 inhibited IL-8 expression in breast cancer cells via directly binding to IL-8 promoter. Moreover, sohlh2 partially attenuated the activation of IL-8 promoter stimulated by TNFα, suggesting the crosstalk between sohlh2 and TNFα/NFκB signaling pathway on IL-8 transcription. Ten E-box motifs were identified in the human IL-8 promoter. bHLH transcription factor can bind to the E-box motifs on the IL-8 promoter and repress its activity [41]. In the future study, the specific E-box motifs bound by sohlh2 are worthy to be identified.

In conclusion, we provide the first evidence that sohlh2 inhibits EMT in breast cancer cells. The underlying mechanism involves direct disruption of IL-8 expression, which further leads to the inhibition of migration, invasion and metastasis of breast cancer cells. Overall, our results strongly support that sohlh2 regulates the motility, invasiveness and metastatic potentials of breast cancer cells by suppressing IL-8 expression.

## MATERIALS AND METHODS

### Cell lines, plasmids and reagents

The human breast cancer cell lines (MCF-7, MDA-MB-231) and human transformed mammary epithelial cells (HBL-100, MCF-10a) used in this study were purchased from the Cell Bank of Type Culture Collection of the Chinese Academy of Sciences (Shanghai, China). The human breast cancer cells were cultured in DMEM medium supplemented with 10% fetal bovine serum (FBS; Gibco, Life Technologies, Grand Island, NY) and 1% penicillin-streptomycin. All cells were grown at 37°C in an atmosphere of 5% CO_2_. All transfections were carried out using Lipofectamine 2000 (Invitrogen, Life Technologies).

The human sohlh2 cDNA was cloned into the pEGFP-N1 vector (Clontech, CA) to generate sohlh2-overexpressing plasmid, pSohlh2. The luciferase reporter plasmid pGL4-IL-8 was constructed by cloning the 770 bp promoter regions of human IL-8 into the promoterless pGL4-basic vector (Promega, Madison, WI) as described previously [40]. The shRNAs were cloned into the GV428 vector (Genechem, Shanghai, China), using the mRNA targeted sequences as follows: Sohlh2: 5′- GC UCCAAUUCCUGACUAAUAC −3′ (pShS1) and 5′- UC UCCUGCCGUUAUGGCCCAGAUUA −3′ (pShS2), IL-8: 5′- GCCAAGGAGUGCUAAAGAA −3′ (pShIL-8). All constructs were verified by sequencing.

Antibodies to Sohlh2, fibronectin and IL-8 were purchased from Abcam Inc. (Cambridge, MA). E-cadherin, N-cadherin and vimentin antibodies were from Cell Signaling Technology (Beverly, MA). β-actin antibody was obtained from Santa Cruz Biotechnology Inc. (Santa Cruz, CA). Human recombinant IL-8 was purchased from Peprotech (Rocky Hill, NJ). TriZol was from Invitrogen. RT-PCR kit was from Thermo Fisher Scientific Inc. (Waltham, MA).

### Wound healing assay

Cells were seeded in 6-well plates at a density of 5 × 10^5^ cells/well. Confluent monolayers of cells were scratched with 10 μL pipette tip to form a gap space of approximate 0.6 mm. Cell debris was removed by PBS wash for three times. Photomicrographs were taken at 0 and 24 h after treatment. Five randomly selected points along each wound were marked, and the horizontal wound widths were measured using Image-Pro Plus 6.0 software. The migrated distance of cells was determined by subtracting the wound width at 24 h from its width at 0 h. Experiments were carried out in triplicate and repeated at least three times.

### Migration assay

Cell migration was determined by using a modified two chamber migration assay with a pore size of 8 mm. For migration assay, 1 × 10^5^ HBL-100 and MDA-MB-231 cells were seeded in serum-free medium in the upper chamber. After 12 h incubation at 37°C, cells in the upper chamber were carefully removed with a cotton swab and the cells that had traversed the membrane were fixed in methanol and stained with leucocrystal violet. The number of migration cells was determined by counting the leucocrystal violet stained cells. For quantification, cells were counted under a microscope in five fields (up, down, median, left, right. ×200).

### Cell invasion assay

Cells transfected with pSohlh2 or sohlh2 shRNAs (pShS1 and pShS2) were cultured at ~80% confluence. Cells were starved in basal medium containing 0.2% bovine serum albumin for 16 h. Matrigel cell invasion assay was carried out using the BD BioCoat Tumor Invasion System (BD Biosciences #354165) as recommended by the manufacturer. 5 × 10^4^ starved cells were seeded into the apical chambers, followed by adding a chemoattractant (basal medium plus 10% FBS) to the basal chambers. After 24 h incubation, cells in the upper chamber were carefully removed with a cotton swab and the cells that had traversed the membrane were fixed in methanol and stained with leucocrystal violet. The number of invasive cells was determined by counting the leucocrystal violet stained cells. For quantification, cells were counted under a microscope in five fields (up, down, median, left, right. ×200).

### Metastasis *in vivo*

For experimental metastasis assay, 1 × 10^6^ stable sohlh2-expressing MDA-MB-231cells (sohlh2 RNAi MCF-7 cells) or their parent cells were injected into 6–8 weeks old female BABL/c nude mice via tail vein. Eight weeks after injection, mice were euthanized, and lungs and livers were removed and subjected to histological examination.

For spontaneous metastasis assay, 5 × 10^6^ Sohlh2 overexpressing MDA-MB-231cells were injected in the forth mammary fat pad of BABL/c nude mice. Lungs were either harvested from recipients at the same time as primary tumors (at a volume of approximately 1,000 mm^3^), 8 weeks after primary tumor resection (500 to 750 mm^3^).

For each sample, all micro-metastases were counted under a light microscope at 10x magnification by an observer blinded to the experimental conditions. Six sections at 50 μm interval were counted per mouse sample. Animal experiments were approved by the Laboratory Animal Ethics Committee of Shandong University and conformed to the legal mandates and national guidelines for the care and maintenance of laboratory animals.

### ELISA

Breast cancer cell lines were transfected with control or pSohlh2 vector. Forty-eight hours after transfection, the vector- and sohlh2-expressing cells were seeded at a density of 1 × 10^5^ cells per well in a 12-well plate and cultured for additional 24 h. IL-8 concentration in culture supernatants was determined using ELISA kit (BD Biosciences) following manufacturer's instructions.

### Patient samples and immunohistochemistry

77 cases of primary human invasive ductal breast cancer specimens, 16 cases of intraductal carcinoma *in situ*, and 25 cases of adjacent tissues to breast cancer were collected from patients at Qilu Hospital and Weifang People's Hospital from 2011 to 2013. The histological characterization and cell differentiation-based breast tumor grading were determined according to the current Union for International Cancer Control (UICC) criteria. The written consent was obtained from all patients and this study was approved by the Medical Ethics Committee of Shandong University.

The tissues were stained according to the standard immunostaining procedure [8]. Briefly, formalin-fixed and paraffin-embedded tissues were sectioned (5 μm). After deparaffinization, sections were immunostained using anti-Sohlh2 and anti-IL-8 at 1:100 dilution. The slides were counterstained with hematoxylin and mounted. Control experiments were performed using non-immune immunoglobulins instead of the specific antibody. The immunostaining images were captured using the Olympus computerized image analysis system.

Each sample of Sohlh2 immunostaining was scored according to the intensity (no staining = 0; weak staining = 1; moderate staining = 2; strong staining = 3) and the percentage (0% = 0; 1–10% = 1; 11–50% = 2; 51–80% = 3; 81–100% = 4) of stained cells. Final immunoreactive scores were determined by multiplying the intensity score with the percentage score, which ranged between 0 and 12 [42, 43]. Sohlh2-positive expression was defined for tumors with Sohlh2-immunoreactive scores from 4 to 12, while Sohlh2-negative expression was defined for tumors with Sohlh2 immunoreactive scores from 0 to 3.

### Quantitative real time-PCR

Total RNA was prepared using TriZol reagent (Invitrogen) following the manufacturer's instructions. Five micrograms of total RNA was reverse-transcribed using the SuperScript II Reverse Transcriptase Kit (Invitrogen). A 25-μL volume reaction consisted of 1 μL reverse transcription product and 10 pM of each primer. The specific primers used for RT-PCR were listed in Supplementary Table S2.

### Western blot

Cells were lysed in RIPA lysis buffer (Santa Cruz). Total 25 μg proteins were separated on a 10% SDS–polyacrylamide gel, and then transferred to polyvinylidene fluoride membranes (Millipore Corp, Billerica, MA). The membranes were probed with appropriate primary antibody at 4°C overnight, followed by incubation with peroxidase-conjugated anti-rabbit (or anti-goat) IgG antibody for 1 hr at room temperature. The interaction was monitored with an enhanced chemiluminescence kit (Amersham Life Sciences Inc., GE Healthcare Life Sciences, Pittsburgh, PA). Anti-β-actin antibody was used to monitor the loading amount.

### RNA sequencing

The 3′ tag DGE libraries were constructed from sohlh2 RNAi MCF-7 cells as described in the Illumina DGE protocol. Total RNA (1–2 μg) was fractionated using oligo-dT magnetic beads to yield poly(A+) mRNA. mRNA bound to the beads was then used as a template for first strand cDNA synthesis primed by oligo-dT and the second strand cDNA was consequently synthesized using random primers. The double strand cDNA is purified with magnetic beads and washed with EB buffer for end-polishing. Sequencing adapters are ligated to the fragments finally. The fragments are purified by magnetic beads and enriched by PCR amplification. The library products are ready for sequencing analysis via Illumina HiSeq^TM^ 2000. RNA sequencing yielded a mean read count of ~12 million reads per sample. After selection of intron-spanning (spliced) RNA reads and exclusion of genes with low coverage, we detected 15382 different protein coding and non-coding RNAs that were used for subsequent analyses. False discovery rate (FDR) ≤ 0.001 and the absolute value of log_2_ Ratio, ≥ 1 were used as the threshold to judge the significance of gene expression difference.

### ChIP assay

ChIP analysis was performed as described previously [11]. Cells stably expressing sohlh2 or control vector were prepared following the guidelines of ChIP assay kit (Upstate Chemicon). Chromatin solutions were precipitated with 30 μL of agars containing anti-sohlh2 antibody (ab101402, Abcam) at 4°C overnight with agitation. For a negative control, rabbit IgG was used to incubate with the supernatant fraction for 1 h at 4°C with rotation. Precipitated DNAs were analyzed by qPCR. The 3 primer pairs for human IL-8 promoters (two primer pairs spanning E-Motifs and one control primer) were as follows: IL-8-1: 5′- GATTGGCTGGCTTATCTTCACC −3′ and 5′- TCCTTATCAAATACGGAGTATGACG −3′(PCR product covering the sequences from −505 to – 152); IL-8-2: 5′-GAAGCAACAGTGGCTGAACCAG −3′and 5′-GAAG TGAGACAATTGTACGTAA −3′(from −1110 to −640); IL-8-con: 5′-TGTGAGCATCAAGGTTAAGTAG −3′and 5′-CTTAGTCAGTTCGGTCCAACACAG −3′(from −2443 to −2183)

### Luciferase reporter assay

5 × 10^4^ MDA-MB-231 cells were plated in 24- well plate and transiently transfected with 200 ng of sohlh2 plasmid, 100 ng of IL-8 promoter firefly luciferase reporter construct and 17 ng of Renilla luciferase plasmid (Promega) using Lipofectamine 2000 (Invitrogen). Luciferase activities were determined 24 hours after transfection using a dual-luciferase reporter assay system (Promega). Results were represented as the ratio of firefly to Renilla luciferase activity and normalized to vector control.

### Statistical analysis

Quantitative data are expressed as mean ± standard deviation (SD). GraphPad Prism (GraphPad Software, San Diego, CA) was used for data analysis. The Student *t* test or one-way analysis of variance (ANOVA) was used to assess significant differences between groups. The Chi-square test was used to analyze the relationship between categorical variables. *P* < 0.05 was considered statistically significant.

## SUPPLEMENTARY MATERIALS TABLES



## Abbreviations

EMT: epithelial-to-mesenchymal transition
Sohlh2: spermatogenesis- and oogenesis- specific basic helix-loop-helix transcription factor2
IL-8: interleukin 8
FBS: fetal bovine serum
ChIP: chromatin immunoprecipitation
