# Supplementary material for: Sohlh2 suppresses epithelial to mesenchymal transition in breast cancer via downregulation of IL-8
Source: Oncotarget. 2016 Jun 30;7(31):49411–24. doi: 10.18632/oncotarget.10355 (PMC5226517; doi:10.18632/oncotarget.10355)
Supplement: Supplementary file 1 [file oncotarget-07-49411-s001.pdf]

Sohlh2 suppresses epithelial to mesenchymal transition in breast cancer via downregulation of IL-8

Supplementary Materials

Supplementary Table S1: Differential gene expression in MCF7-sohlh2 RNAi and control cells

#GeneID	Symbol	Control	Sohlh2 RNAi	log2 Ratio (RNAi/Control)	*P*-value	FDR
3576	IL8	10	134	3.660325115	1.42E-27	1.89E-25
3280	HES1	17	74	2.038154544	3.85E-09	8.91E-08
768	CA9	43	170	1.899290201	1.12E-17	8.06E-16
79094	CHAC1	58	225	1.871964216	1.64E-22	1.64E-20
1649	DDIT3	128	412	1.602664547	1.85E-32	3.31E-30
1999	ELF3	29	80	1.38011112	3.25E-06	4.21E-05
602	BCL3	53	146	1.378068124	3.00E-10	8.66E-09
8614	STC2	237	618	1.298883799	2.07E-35	4.37E-33
51129	ANGPTL4	36	91	1.254033659	3.94E-06	4.97E-05
7745	ZNF192	41	14	–1.634033063	9.91E-05	0.0008595
360023	ZBTB41	78	27	–1.614350697	8.89E-08	1.61E-06
170959	ZNF431	60	21	–1.598409153	3.32E-06	4.29E-05
79598	CEP97	59	21	–1.574161607	5.13E-06	6.30E-05
349408	TLR8-AS1	1132	407	–1.559609239	1.12E-88	1.14E-85
389136	VGLL3	178	66	–1.515175292	1.03E-14	5.63E-13
6526	SLC5A3	115	43	–1.503061276	6.70E-10	1.85E-08
222236	NAPEPLD	48	18	–1.498873479	7.46E-05	0.0006699
2354	FOSB	309	117	–1.484934288	9.23E-24	1.04E-21
134957	STXBP5	71	27	–1.478695597	1.74E-06	2.39E-05
257218	SHPRH	53	21	–1.419439012	6.35E-05	0.00058057
5166	PDK4	60	24	–1.405764075	2.36E-05	0.00024547
27332	ZNF638	423	170	–1.398958897	2.44E-29	3.55E-27
2957	GTF2A1	57	23	–1.393164038	4.29E-05	0.00041253
56204	FAM214A	64	26	–1.383396262	1.59E-05	0.00017228
80314	EPC1	73	30	–1.366769943	4.88E-06	6.05E-05
7403	KDM6A	58	24	–1.356854475	5.22E-05	0.00048727

Supplementary Table S2: Primer sequences for qPCR

Genes	Primers
sohlh2	Forward 5′-CAACATGTCCCTCAAACACTG-3′
	Reverse 5′-GCCCATTGCCATTCCTTAAAG-3′
IL8	Forward 5′-AGTGAGCTCATTGGCTGGCTTATCTTC-3′
	Reverse 5′-AGTAAGCTTGTTTCTTCCTGGCTCTTG -3′
E-cadhenrin	Forward5′-CGAGAGCTACACGTTCACGG-3′
	Reverse 5′-GGGTGTCGAGGGAAAAATAGG-3′
N-cadherin	Forward 5′-CAGAATCAGTGGCGGAGATC-3′
	Reverse 5′-CAGCAACAGTAAGGACAAACATC-3′
Fibronectin	Forward 5′-CTGAGACCACCATCACCATTAG-3′
	Reverse 5′-GGGCTCGCTCTTCTGATTATT-3′
Vimentin	Forward 5′-CGTGAATACCAAGACCTGCTC-3′
	Reverse 5′-GGAAAAGTTTGGAAGAGGCAG-3′
GAPDH	Forward 5′-ATCTTCCAGGAGCGAGACCCC-3′
	Reverse 5′-TCCACAATGCCAAAGTTGTCATGG-3′
